# Critically ill patients in emergency department may be characterized by low amplitude and high variability of amplitude of pulse photoplethysmography

**DOI:** 10.1186/1757-7241-21-48

**Published:** 2013-06-24

**Authors:** Jussi Pirneskoski, Veli-Pekka Harjola, Petri Jeskanen, Lari Linnamurto, Simo Saikko, Jouni Nurmi

**Affiliations:** 1 Department of Anesthesia and Intensive Care, Helsinki University Central Hospital, Helsinki, Finland; 2 Division of Emergency Care, Department of Medicine, Helsinki University Central Hospital, Helsinki, Finland; 3 Saimaa University of Applied Sciences, Lappeenranta, Finland

## Abstract

**Background:**

The aim of the present pilot study was to determine if pulse photoplethysmography amplitude (PPGA) could be used as an indicator of critical illness and as a predictor of higher need of care in emergency department patients.

**Methods:**

This was a prospective observational study. We collected vital signs and one minute of pulse photoplethysmograph signal from 251 consecutive patients admitted to a university hospital emergency department. The patients were divided in two groups regarding to the modified Early Warning Score (mEWS): > 3 (critically ill) and ≤ 3 (non-critically ill). Photoplethysmography characteristics were compared between the groups.

**Results:**

Sufficient data for analysis was acquired from 212 patients (84.5%). Patients in critically ill group more frequently required intubation and invasive hemodynamic monitoring in the ED and received more intravenous fluids. Mean pulse photoplethysmography amplitude (PPGA) was significantly lower in critically ill patients (median 1.105 [95% CI of mean 0.9946-2.302] vs. 2.476 [95% CI of mean 2.239-2.714], P = 0.0257). Higher variability of PPGA significantly correlated with higher amount of fluids received in the ED (r = 0.1501, p = 0.0296).

**Conclusions:**

This pilot study revealed differences in PPGA characteristics between critically ill and non-critically ill patients. Further studies are needed to determine if these easily available parameters could help increase accuracy in triage when used in addition to routine monitoring of vital signs.

## Background

Triage is a method adopted in to the daily clinical practice in the 1960’s from military medicine to answer the need for sorting the increasing amount of patients in emergency departments (EDs) [1]. Multiple different triage instruments have been developed to aid in the process and are widely used around the world e.g. Emergency Severity Index (ESI) [2], Soterion Rapid Triage System (SRTS) [3], Canadian Triage and Acuity Scale (CTAS) [4] and modified Early Warning Score (mEWS) [5]. Of these mEWS is simple taking into account only the vital functions of the patient whereas e.g. ESI includes also information on current resources and symptoms of the patient. mEWS (Table 1) has been shown to predict need for higher intensity of care and risk of death [5,6]. The application of different triage instruments is varied in both ED and ward settings and no international consensus exists supporting the use of a single scoring system.

**Table 1:** Modified early warning score

**Score**	**3**	**2**	**1**	**0**	**1**	**2**	**3**
Systolic BP, mmHg	< 70	71-80	81-100	101-199		≥ 200	
Heart rate, min^-1^		< 40	41-50	51-100	101-110	111-129	≥ 130
Respiratory rate, min^-1^		< 9		9-14	15-20	21-29	≥ 30
Temperature, °C		< 35		35-38.4		≥ 38.5	
AVPU score				Alert	Reacting to voice	Reacting to pain	Unresponsive

Peripheral photoplethysmographic pulse wave is dependent on peripheral perfusion and can either be described as photoplethysmographic pulse wave amplitude (PPGA, the total height of the photoplethysmographic pulse wave) [7,8] or as perfusion index (PI, the relation of the pulsatile component to the non-pulsatile component of the photoplethysmographic pulse wave) [9]. Plethysmographic wave amplitude is lower in critically ill patients with impaired hemodynamics [10] and in critically ill neonates [9]. It also decreases as sequence of pain [11]. Respiratory related variation in PPGA increases in hypovolemic conditions and can be used, with some limitations, to evaluate the volume responsiveness [12-16]. So far pulse photoplethysmographic indices have been mainly studied on mechanically ventilated patients in operative or intensive care settings, but some work has been published on spontaneously breathing patients as well [9,10,14,17-19].

Since pulse oximeters are already widely available in emergency departments, we investigate if pulse photoplethysmography could be used in an emergency department as an easy-to-use triage tool. The aim of this pilot study was to investigate if the pulse photoplethysmography derived indices would be able to help to discriminate critically ill patients with need for higher intensity care in the emergency department.

## Methods

The prospective observational cohort study was approved by the Ethics Committee of Medicine in Helsinki Uusimaa Hospital District and was performed without external funding. No written consent was required by the Ethics Committee. All patients over 18 years of age admitted to the ED for any reason during 72 hour data collection period were included in the study. Patients with incomplete data were excluded from the final analysis. The study was conducted in a tertiary referral university teaching hospital ED covering following specialties: general medicine, respiratory medicine, neurology, gastroenterological surgery, vascular surgery and thoracic surgery. The study did not affect the treatment or triage classification of the patients. Only the two research nurses responsible for the data recording were aware of the pulse photoplethymography values.

Basic vital signs including blood pressure, pulse rate, respiratory rate, capillary refill (over or under 2 seconds), oxygen saturation (SpO_2_), level of consciousness (on a 4 level AVPU scale: alert, responds to voice, responds to pain, unresponsive), and body temperature (both core and peripheral, from tympanum and index finger, respectively) were measured and recorded on arrival to ED by either of the two research nurses. Possible use of vasoactive medications before data collection was recorded.

The pulse photoplethysmograph signal was collected using an AS/3 monitor (GE Healthcare, Little Chalfont, United Kingdom) for one minute from an index finger during the triage within ten minutes of presentation to the ED immediately before measuring the basic vital signs. Nail polish was removed with acetone if present. Data was recorded with Collect S/5 software, version 4.0 (GE Healthcare, Little Chalfont, United Kingdom) and PPGA was automatically averaged every 10 seconds as previously described [11]. No calibration for the hardware or software was necessary during the measurements. The research nurses received hands-on training in the use of the equipment and their performance in using it was frequently evaluated by one of the researchers.

To describe the intensity of care needed in the ED, we registered interventions performed (invasive hemodynamic monitoring and intubation) and total volume of intravenous fluid received. Based on patient records we collected data on highest level of follow-up care during the hospital stay [discharged from ED, ward, high dependency unit (HDU), intensive care unit (ICU), operating theatre (OR), transfer to another hospital] as well as survival to hospital discharge.

Maximum, minimum and mean of PPGA (PPGAmax, PPGAmin, PPGAmean) were determined for each patient from collected data after the data collection period. To approximate variation in the PPGA signal, PPGAvar was derived from the equation used by Broch et al. [13] to determine pleth variability index (PVI): PPGAvar = (PPGAmax – PPGAmin) / PPGAmax. To classify critically ill and non-critically ill patients, modified Early Warning Score (mEWS) was calculated from the basic vital functions for each patient and mEWS > 3 was used as cut-off as shown to predict higher requirement for intensive care in an earlier study [20]. MEWS as a proven predictor of death and critical illness [5,6] was used as a surrogate due to the pilot nature and limited number of patients in the study and thus death as an outcome was deemed to be unlikely to reach statistical significance.

Statistical analysis was performed using GraphPad Prism, version 5.0d (GraphPad Software Inc., San Diego, CA, USA). We determined standard deviations, interquartile ranges, mean values and 95% confidence intervals (CI) for different variables. The variables were analyzed for normality using D’Agostino & Pearson test. Because all other data collected except for heart rate were not normally distributed, we analyzed the data using Mann–Whitney test for other variables and unpaired T test for heart rate. For correlation analysis we used Spearman correlation where applicable. We also calculated receiver operating characteristic (ROC) curves for the different photoplethysmography variables to detect criticall illness. P-value of < 0.05 was considered statistically significant.

## Results

During the data collection period a total of 251 patients admitted to the ED. Of these, sufficient data for final analysis was available for 212 (84.5%) patients. Characteristics of the patients are shown in Table 2.

**Table 2:** Characteristics of critically ill (modified early warning scrore, mEWS > 3) and non-critically ill (mEWS ≤ 3) patients

	**mEWS > 3 (N = 18)**	**mEWS ≤ 3 (N = 194)**	**p-value**
Characteristics			
Age (years, median, IQR, 95% CI)	65.49 (35.32-76.64, 47.12–68.50)	57.15 (40.01-71.25, 53.65-59.06)	0.7072
Sex (males, %, 95% CI)	5 (27.78, 12.17-51.20)	104 (53.61, 46.59-60.49)	0.0478
Systolic BP (mmHg, median, IQR, 95% CI)	108 (95–139, 98.14-134.1)	131 (119–151, 133.0-139.7)	0.0013
Heart rate (min^-1^, median, IQR, 95% CI)	103 (75–117, 87.47-115.0)	75 (64–84, 72.53-76.53)	0.0008
Respiratory rate (min^-1^, median, IQR, 95% CI)	20 (14–24, 16.64-22.65)	16 (14–20, 16.87-18.14)	0.1156
Tympanic temperature (°C, median, IQR, 95% CI)	36.5 (36.0-37.8, 35.43-37.57)	36.8 (36.4-37.1, 36.71-36.87)	0.4290
Temperature difference between tympanum and finger (°C, median, IQR, 95% CI)	8.5 (4.9-10.5, 6.303-9.486)	6.7 (4.8-9.3, 6.560-7.352)	0.2290
mEWS (points, median, IQR)	5 (4–5)	1 (1–2)	< 0.0001
Death during hospital stay (n, %, 95% CI)	0 (0, 0–20.67)	1 (0.515, 0.01-3.16)	> 0.9999
Required intensive care during hospital stay (n, %, 95% CI)	4 (22.22, 8.47 – 45.75)	14 (7.216, 4.26-11.84)	0.0524
Transferred to OR (n, %, 95% CI)	5 (27.78, 12.17-51.20)	12 (6.186, 3.47-10.60)	0.0014
Transferred to another hospital (n, %, 95% CI)	6 (33.33, 16.10-56.43)	20 (10.31, 6.71-15.45)	0.0049
Transferred to a ward (n, %, 95% CI)	4 (22.22, 8.47-45.75)	53 (27.32, 21.52-34.00)	0.6261
Discharged from ED (n, %, 95% CI)	6 (33.33, 16.10-56.43)	97 (50.00, 43.03-56.97)	0.1648
Intubated in the ED (n, %, 95% CI)	4 (22.22, 8.47-45.75)	0 (0.0, 0.0-2.34)	< 0.0001
Invasive hemodynamic monitoring in the ED (n, %, 95% CI)	6 (33.33, 16.10-56.43)	6 (3.093, 1.27-6.74)	< 0.0001
Intravenous fluid replacement (ml, median, IQR, 95% CI)	1560 (763–4050, 1246–4022)	125 (0–1090, 564–888)	< 0.0001

*IQR* interquartile range, *CI* confidence interval.

We recorded the use of vasoactive drugs from 15 minutes before the patient was admitted to the ED. Only two patients received vasoactive drugs: one patient norepinephrine and one patient glyceryl trinitrate, the amount was not recorded.

**Figure 1:**
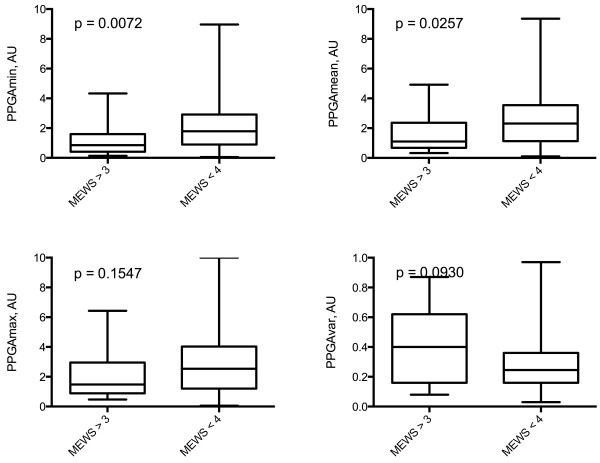
**PPGA variables in critically ill and non-critically ill patients.** AU, arbitrary units; PPGA, pulse photoplethysmography amplitude.

Patients with mEWS >3 needed higher level of care in the ED as well as larger amount of fluid replacement (Table 2). Of the pulse photopletysmography variables PPGAmin and PPGAmean were lower in critically ill patients with mEWS score > 3 (Figure 1).

PPGAmin and PPGAvar significantly correlated with mEWS scores (r = −0.1571, p = 0.0221, 95% CI = −0.2895 - -0.0188 and r = 0.1816, p = 0.0080, 95% CI = 0.0440-0.3125, respectively) and with total volume of intravenous fluids received (r = −0.1379, p = 0.0459, 95% CI = −0.2721 - -0.0015 and r = 0.1501, p = 0.0296, 95% CI = 0.0110-0.2836, respectively).

We also found a significant correlation between PPGAmin (r = −0.6344, 95% CI = −0.7111 - -0.5428), PPGAmax (r = −0.6514, 95% CI = −0.7251 - -0.5629) and PPGAmean (r = −0.6685, 95% CI = −0.7392 - -0.5833, p < 0.0001 for all) and temperature difference between the finger and the tympanum, surrogate of peripheral perfusion.

The ROC curves were calculated for pulse photoplethysmography variables to differentiate between the critically ill and non-critically ill groups. AUC values were 0.6894 for PPGAmin (p = 0.0079), 0.6018 for PPGAmax (p = 0.1534), 0.6582 for PPGAmean (p = 0.0265) and 0.6198 for PPGAvar (p = 0.0928).

## Discussion

We have demonstrated that in unselected ED patients PPGAmin and PPGAmean are lower in critically ill patients determined by mEWS score > 3. Lower PPGAmin and higher PPGAvar also correlated with higher amounts of intravenous fluids received which is in line with volume depletion effects on PPG described in previous studies on healthy volunteers [18,19]. Due to the limits of the methodology used in data collection, no true beat to beat variation was calculable of the data. Thus we used the derived variable PPGAvar to approximate the amount of variability in the photoplethysmographic signal which is to a large degree but not solely induced by respiratory effect on peripheral volume status.

Due to the pilot nature of the study and the challenges of collecting data in the ED we collected data on a limited number of patients. To facilitate data analysis we compared PPGA variables through a previously validated surrogate, mEWS. The feasibility of the surrogate in the current study for critical illness, mEWS with cut-off of 3, was confirmed by higher rate of advanced intensive care procedures performed and considerably greater total volume of intravenous fluids received in this group. This scoring system has also been shown to predict mortality and need of intensive care in previous studies [5,6,20]. However, in our study, no differences in ICU admissions or mortality were observed, probably due to the small sample size and the fact that 12% of the patients were transferred to another hospital from the ED. The study protocol was also designed so that the measurements should not hinder the treatment of patients. Thus we may have unintentionally excluded the most critically ill patients from the study.

Peripheral circulation and peripheral temperature are known to decrease in haemodynamic shock. This is caused by increased levels of catecholamines and sympathetic response [21,22]. Difference between peripheral and core temperature has been studied since the 1960’s [23-25]. Peripheral vasoconstriction also induces changes in pulse photoplethysmographic variables. Strong correlation between PPGA and the temperature gradient has been reported [10]. In this study, we were also able to clearly demonstrate lower PPGA values in patients with higher difference between core and peripheral temperatures. This suggests that PPGA could possibly be used in addition to core-peripheral temperature difference to assess the hemodynamic status of patients.

Based on the findings of the current study, studied photoplethysmographic variables cannot be used as sole method of differentiating critically ill patients from non-critically ill in the ED. Still they could be applied to current triage instuments such as mEWS easily since photoplethysmograph is readily available and it’s already used for measurements routinely.

Because of the pilot nature of the current study, the number of patients was limited and the results should be validated in a larger study. Limited study population also could have affected the ability of the study to detect differences in rare outcomes such as in-hospital death. The current study also had a number of other limitations. We were unable to fully register the effect of breathing cycle on PPGA variables and thus peripheral blood volume, since PPGA was automatically averaged by 10-second intervals. We also gathered the photoplethysmograph signal for only one minute on arrival and thus lost the possibility of further analysis of the signal including e.g. frequency domain analysis [26]. This time period was chosen because we did not want to hinder the treatment of the patients and because we found it unreasonable to perform measurements taking long amount of time in the ED setting. The use of vasoactive drugs potentially affects the interpretation of PPGA variables by modifying the peripheral vasodilation, but only two patients received such medications, so this was not likely to cause a significant error in the analysis.

## Conclusions

It appears that pulse photoplethysmography amplitude (PPGA) might provide information which could be useful in addition to existing triage instruments to enhance the triage specificity in unselected referral emergency department patients. PPGAmin and PPGAmean are both lower in patients with mEWS score > 3 than in patients with mEWS score ≤ 3. Lower PPGAmin and higher PPGAvar also correlated with higher amount of intravenous fluids received. The results in this pilot study are only suggestive and warrant further research and validation in larger study population.

## Competing interests

The authors of this study report no competing financial or non-financial interests.

## Authors’ contributions

JP carried out the statistical analysis and drafted the manuscript. PJ and LL collected the data and participated in the design of the study. SS and VPH participated in the design and coordination of the study. JN conceived of the study, and participated in its design and coordination and helped to draft the manuscript. All authors read and approved the final manuscript.
